# Early Conversion to Tacrolimus *Vs* Cyclosporine Continuation in Normally Functioning Kidney Allograft: A Single-Center Study

**DOI:** 10.22037/ijpr.2020.113220.14174

**Published:** 2020

**Authors:** Laya Azizzadeh, Seyed Amirhossein Fazeli, Farshad Hashemian, Sanaz Dehghani, Seyedeh Samaneh Ahmadi, Gholamreza Pourmand

**Affiliations:** a *Department of Clinical Pharmacy, Faculty of pharmacy, Tehran Medical Sciences, Islamic Azad University, Tehran, Iran. *; b *Nephrology Research Center, Tehran University of Medical Sciences, Tehran, Iran. *; c *Urology Research Center, Sina Hospital, Tehran University of Medical sciences, Tehran, Iran. *; d *Rajaie Cardiovascular, Medical, and Research Center, Iran University of Medical Sciences, Tehran, Iran.*

**Keywords:** Calcineurin inhibitors, Immunosuppressive, Graft, Kidney, Transplantation

## Abstract

This study evaluated the effectiveness of early pre-emptive conversion from cyclosporine to tacrolimus in kidney transplant patients with normal graft function and in the absence of adverse effects of the initial cyclosporine. A historical cohort study of 166 patients who received deceased-donor kidney transplant between 2011 to 2017 was conducted. All the patients had been treated with cyclosporine (Sandimmune®) during their immediate post-transplantation period. At the time of hospital discharge, the patients were divided into 2 groups: patients with continued cyclosporine (Sandimmune®) treatment (n = 125) and the patients whose treatments converted from cyclosporine to tacrolimus (Prograf®) at discharge (n = 41). The 1-year graft function (*p* = 0.074), acute rejection (*p* = 0.566), and graft loss (*p* = 0.566) were not significantly different between two groups. The patients on tacrolimus had lower levels of cholesterol (*p* = 0.002) and diastolic blood pressure (*p* = 0.015). The long-term follow-up showed no significant difference in graft loss (*p* = 0.566). The patients received tacrolimus had higher all-cause mortality within the first year posttransplantation (*p* = 0.002) as well as long-term follow-up (*p* = 0.001). The continuation of initial cyclosporine might be a good option when the graft function is acceptable and the adverse effects are absent.

## Introduction

Long-term institution of immunosuppressive agents is crucial for optimal function of kidney allograft (1). Since many years ago, it is well known that calcineurin inhibitors (CNIs) could be as a mainstay immunosuppressive in kidney transplantation. Cyclosporine (CsA) and tacrolimus (TAC) are currently the most widely used essential immunosuppressives for prevention of acute rejection following kidney transplantation (2).

In renal transplant patients, long term treated with TAC resulted in a lower renal resistance index and less need for antihypertensive compared with CsA and has been associated with less rejection and better kidney function (3,4). It has been suggested that the cardiovascular risk profile of TAC is more favorable than that of CsA as it has less propensity to cause hyperlipidemia and hypertension (5, 6) and several trials reported an increased graft survival in patients using TAC as initial immunosuppressive treatment (2,7,8). In contrast CsA was associated with superior glycemic profile, diminished incidence of BK virus nephropathy (9,10) and lower association with posttransplant lymphoproliferative disorder (PTLD) (11).

Given aforementioned safety and efficacy superiority, the current CNI of choice for kidney posttransplant maintenance immunosuppression is TAC (12). Conversion from CsA to TAC is a favorable policy following development of chronic allograft nephropathy (CAN) or in cases when relevant adverse effects are evolved, for example, CNI change from CsA to TAC in CAN was associated with ameliorated graft function (13,14). Furthermore, Woodle *et al*. had considered TAC as a suitable alternative treatment for prior CsA therapy in the context of renal allograft acute rejection (15). However, there are considerable controversies in terms of patients’ and graft outcomes as well as resultant adverse effects following such conversion in different studies (1, 4, 6, 13, 14, 16-20).

To our best of knowledge there are limited studies evaluating efficacy of late conversion to TAC *vs* continuation of CsA in stable functioning kidney grafts. Artz *et al*. and Plischke *et al*. demonstrated improved graft function in the patients who received switched TAC after one year following transplantation (19, 20). Late conversion to TAC was associated with better cardiovascular risk profile including blood pressure and lipid profile in recipients of stable function kidney graft. However, the conversion to TAC failed to improve incidence of new-onset of diabetes mellitus after transplantation (NODAT) and patient survival (19).

Conversion from CsA to TAC in early post transplantation period is not well-documented. This study evaluated the effectiveness of early de novo conversion from cyclosporine to tacrolimus in kidney transplantation patients with normal graft function and in the absence of adverse effects of the initial cyclosporine.

## Experimental

*Study characteristics*

This was a single-center, historical cohort study conducted at Sina hospital, Tehran, Iran in 2019. The study evaluated the efficacy of early pre-emptive conversion from CsA to TAC at time of hospital discharge in the patients with normally functioning graft in the absence of cyclosporine-related adverse effects. The patients received deceased-donor kidney allografts from March 2011 to March 2017. 

The primary endpoint was to evaluate efficacy of early pre-emptive conversion from CsA to TAC in terms of short-term graft function (*i.e.* estimated glomerular filtration rate [eGFR] calculated by CKD-EPI equation), the incidence of biopsy-proven acute rejection (BPAR), graft loss, and rate of all-causes patients’ mortality, within 1 year after kidney transplantation. Moreover, through a long-term follow-up, the graft loss and all-causes mortality were also evaluated within 2 to 8 years following the transplantation. The secondary endpoints were effects of aforementioned conversion on laboratory parameters such as complete blood count, serum electrolytes, liver function tests and serum creatinine as well as cardiovascular risk factors such as blood pressure, lipid profile and incidence of new-onset of diabetes after transplantation (NODAT) within first year post transplantation. The source of relevant data was the patients’ medical profiles including all clinical and laboratory information. To limit any potential bias, a double-blinded method of data collection was made. In addition, all the laboratory examinations were performed in a single-center. Furthermore, all clinical assessments such as auscultatory sphygmomanometry and weight measurement were done exclusively by a single experienced examiner.

The study was performed in accordance to the Declaration of Helsinki and approved by the Iranian National Committee for Ethics in Biomedical Research (IR.IAU.PS.REC.1398.066; https://ethics.research.ac.ir/ProposalView.php?id = 69575). The written informed consent had been taken from all the patients prior to study. 

*Cohort population and study protocol*

**Figure 1:**
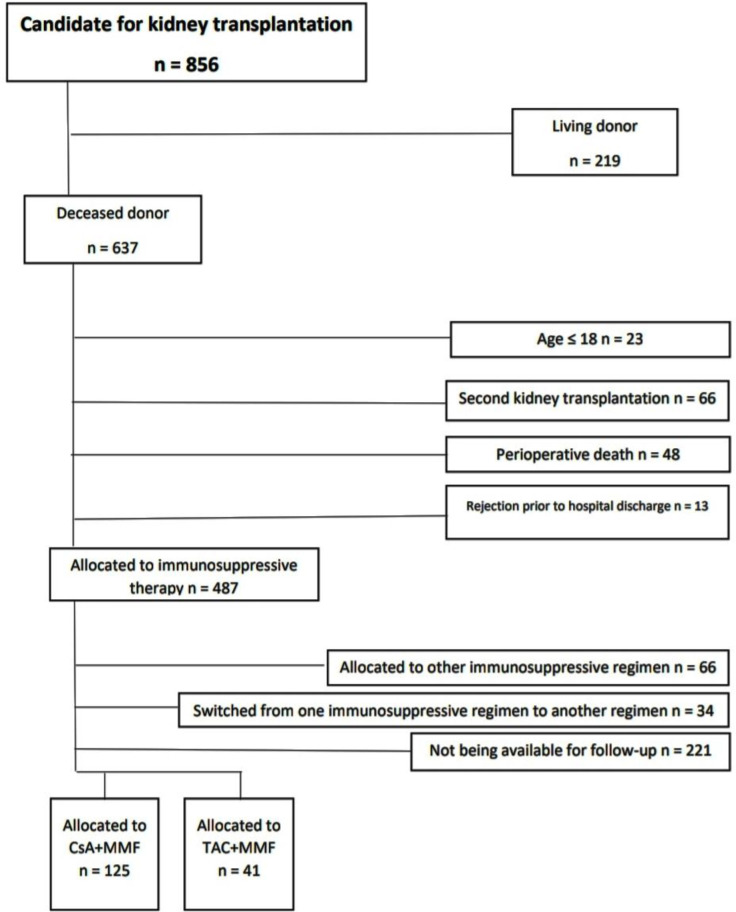
Diagram of the study

The study protocol is outlined in Figure 1. All the patients who received a kidney allograft from March 2011 to March 2017 at Sina Hospital, Tehran, Iran were eligible for enrolling into the study. Following consecutive enrollment, the participants who had exclusion criteria were excluded. The exclusion criteria were living donation, second kidney transplantation, age less than 18, rejection prior to hospital discharge, perioperative death, allocation to miscellaneous immunosuppressive regimen, switching from one immunosuppressive regimen to another regimen during follow-up, and not being available for follow-ups. The final included patients categorized into 2 groups: TAC group *vs* CsA group, described below. The individuals of the study groups were matched according to age, gender, cause of end-stage renal disease and pre-transplantation weight as well as other clinical and laboratory parameters measured prior to transplantation, such as prevalence of diabetes mellitus and hypertension.

All included patients showed low panel-reactive antibodies (PRA) ≤ %20. However, given lack of universal patients’ HLA-typing and virtual or physical cross-matching with the deceased donors, all transplantations had been considered as of high immunological risk and the patients received anti-thymocyte globulin (ATG) 1 mg/kg as induction therapy at time of surgery. In addition, all the patients received pulsed intravenous methylprednisolone (500mg), single dose of oral cyclosporine (Sandimmune®;6-7mg/kg) and mycophenolate mofetil (Cellcept®;3mg/kg) at 4-6 h prior to transplantation. During 7-10 days of hospitalization, ATG, cyclosporine (Sandimmune®), and mycophenolate mofetil (Cellcept®) were continued for all the patients; pulsed methylprednisolone was repeated as needed up to twice again; and daily oral prednisolone (1mg/kg) was started in the 4^th^ day after transplantation for all the patients. At time of hospital discharge, the patients were categorized into 2 groups: The TAC group including patients whose CNI treatment converted from initial CsA to TAC (Prograf®; 0.1 mg/kg/day divided BID) at the hospital discharge and the CsA group including patients whose initial treatment with cyclosporine (Sandimmune®; 6-7 mg/kg/day divided BID) had been continued. The patients of the both groups received mycophenolate mofetil (MMF) and prednisolone as well as CNIs. All the patients were followed up to 12 months after the conversion with regular monthly clinical and laboratory examination. In addition, the patients’ medical documents were reviewed for long-term follow-up. The controlled blood pressures were at < 130 mmHg systolic and < 80 mmHg diastolic as defined by KDIGO guidelines 2009 (12). The NODAT was defined whenever fasting plasma glucose (FPG) was ≥ 126 mg/dL on at least two different days as specified by American Diabetes Association 2017. The fasting was considered as the absent caloric intake of at least 8 h.

*Statistical analysis*

The categorical variables of the study groups, expressed as frequencies and percentage, were compared between the groups by the Chi-square test. Quantitative variables were reported as mean ± standard deviation and were compared using independent sample *t*-test. Normality of the data was checked by skewness histogram and Kolmogorov–Smirnov test. Unadjusted and adjusted effects of therapeutic modalities on time-dependent alteration of several clinical and laboratory parameters were examined by Random Intercept Mixed-effects model. The results were reported as beta-coefficient and 95% confidence interval. The statistical level of significance was defined *P* < 0.05. Statistical analyses were performed using SPSS software of version 23 and STATA version 15.1.

## Results

According to the study diagram outlined in Figure 1, all the patients (n = 856) who received a kidney allograft from March 2011 to March 2017 at Sina Hospital, Tehran, Iran enrolled to the study. Among the patients enrolled consecutively to study, 690 patients were excluded according to our exclusion criteria as follows: living donors (n = 219), second kidney transplantation (n = 66), age less than 18 (n = 23), rejection prior to hospital discharge (n = 13), perioperative death (n = 48), allocation to miscellaneous immunosuppressive regimen (n = 66), switching from one immunosuppressive regimen to another regimen during follow-up (n = 34), and not being available for follow-ups (n = 221). Consequently, 166 patients were included in the study and then categorized into 2 study groups (n = 125 for CsA group; n = 41 for TAC group). As shown in Table 1, the patients switched to TAC and the patients continued on CsA had no statistically significant difference in terms of basal characteristics such as age (*p* = 0.199), sex (*p* = 0.644) and frequency of underlying DM (*p* = 0.697) and HTN (*p* = 0.231). The most frequent cause of ESRD in both groups was HTN and there was no significant difference in causes of ESRD (*p* = 0.107). Furthermore, the patients’ metabolic panels of two group *i.e.* FBS (*p* = 0.999), Liver function tests (*e.g. p *= 0.346 for ALT), and lipid profiles (*e.g. p = *0.679 for HDL) were not significantly different prior to kidney transplantation. The patients whose medications were converted from CsA to TAC compared with those maintained on CsA showed no significant difference in pre-transplantation systolic (*p* = 0.731) and diastolic blood pressure (*p* = 0.999) as well as mean arterial pressures (MAP) (*p* = 0.858). The mean patients’ serum creatinine of both groups were not significantly different indicating that there was no superior pre-transplantation nutritional states (*p* = 0.090). 

At the time of hospital discharge, the graft functions were not significantly different between the two groups (*p* = 0.228). Indeed, the early baseline graft functions prior to CNI change were acceptable and no differential results confounding the follow-up graft functions were found. The doses of daily initial immunosuppression with CsA (*p* = 0.169) cumulative pulsed methylprednisolone (*p* = 0.490) as well as the oral daily glucocorticoid were not significantly different (*p* = 0.111). However, the patients on continued CsA have received higher daily doses of MMF prior to the hospital discharge (1637 ± 379.02 *vs* 1465 ± 354.8 mg/day; *p* = 0.011).

The graft and patients’ outcome following one year of kidney transplantation as outlined in Table 2. Mean CNI dose in patients received TAC and those patients maintained on CsA were 4.61 ± 1.55 mg/day and 224.9 ± 35.8 mg/day, respectively. The patients on CsA received higher doses of MMF (1494.71 ± 304.73 *vs *1371 ± 193.8 mg/day; *p* = 0.003). Given significantly lower mean dose of daily maintenance prednisolone in the patients received TAC, the steroid-sparing effect of TAC were found (17.7 ± 4.1 *vs* 15.8 ± 4.4 mg/day; *p* = 0.013). No statistically significant difference in serum creatinine (*p* = 0.165), creatinine clearance (*p* = 0.783) and estimated GFR (*p* = 0.074) were found in the patients receiving switched TAC compared to patients on continued CsA. Hence, no superior efficiency of either CNIs on short-term graft function (*p* = 0.074) was demonstrated. Furthermore incidence of BPAR (*p* = 0.566) and graft loss (*p* = 0.566) within one-year posttransplantation period was not significantly different between the study groups. Of note, the patients treated with TAC showed the significantly higher rate of all-causes mortality within the same period (*p* = 0.002). The incidences of NODAT (*p* = 0.120) and new-onset hypertension (*p* = 0.491) were not significantly different between two groups. MAP (*p* = 0.413) and percentage of the patients with controlled blood pressure (*p* = 0.601) showed no significant difference. However, the most frequently-used antihypertensive medications in the patients receiving TAC were ACEI/ARB while non-ACEI/ARB antihypertensive were reported in the case of patients on CsA. The percentage of hypertensive patients receiving no antihypertensive were lower in the TAC groups (*p* = 0.042). 

As outlined in table 2, long-term rates of biopsy proven acute rejection and graft loss were not significantly different with median follow-up of 5 years (2 to 8 Yrs. ranged) (*p* = 0.566). The patients who received TAC showed higher rates of long-term all-causes mortality (*p* = 0.001).

**Figure 2:**
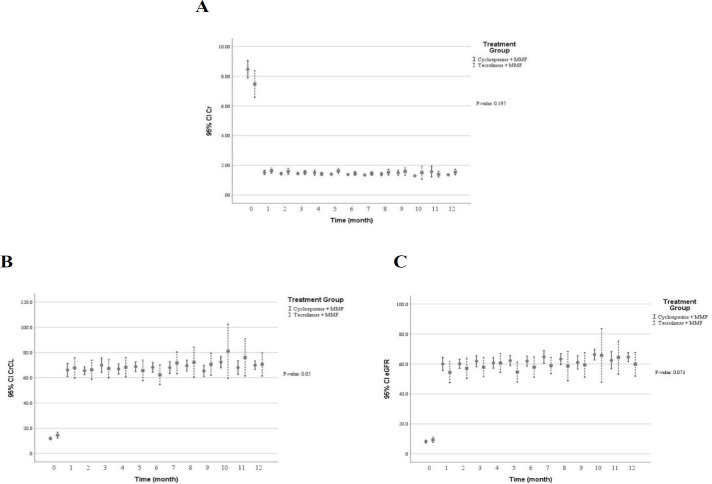
The graft function within the first year after kidney transplantation in the patients received tacrolimus *vs* cyclosporine. (A) serum creatinine (mg/dL) (B) serum creatinine clearance (ml/min) (C) estimated Glomerular Filtration Rate (ml/min). The multivariable predictive analysis showed lower eGFR & CrCL following conversion to tacrolimus in comparison to continuation of cyclosporine therapy. However, the finding was not statistically significant. (*p* ≥ 0.05); MMF: mycophenolate mofetil

**Figure 3:**
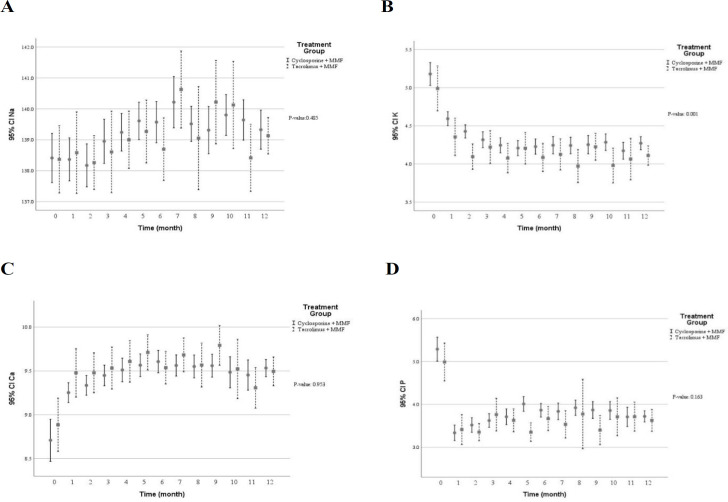
The biochemical profile within the first year after kidney transplantation in the patients received tacrolimus vs cyclosporine (A) serum sodium concentration (mEq/L) (B) serum potassium concentration (mEq/L) (C) serum calcium concentration (mg/dL).(D) serum phosphorus concentration (mg/dL). According to the multivariable predictive analysis, treatment with tacrolimus were associated with significantly higher levels of serum potassium. (*p* ˂ 0.05); MMF: mycophenolate mofetil

**Figure 4:**
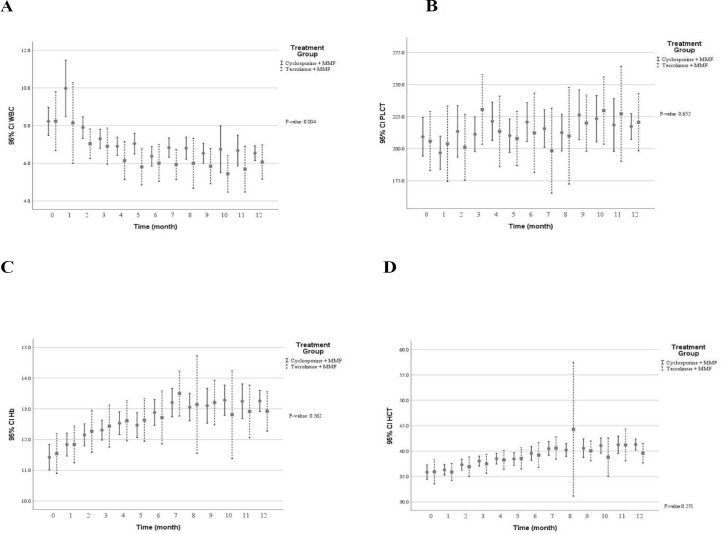
The blood cells profile within the first year after kidney transplantation in the patients received tacrolimus *vs* cyclosporine (A) white blood cells number (×10ˆ3/μL) (B) blood platelets number (×10ˆ3/μL) (C) blood hemoglobin concentration (gr/dL) (D) hematocrit percentage (%). According to the multivariable predictive analysis, treatment with tacrolimus were associated with significantly lower levels of white blood cells. (*p* ˂ 0.05); MMF: mycophenolate mofetil

**Figure 5:**
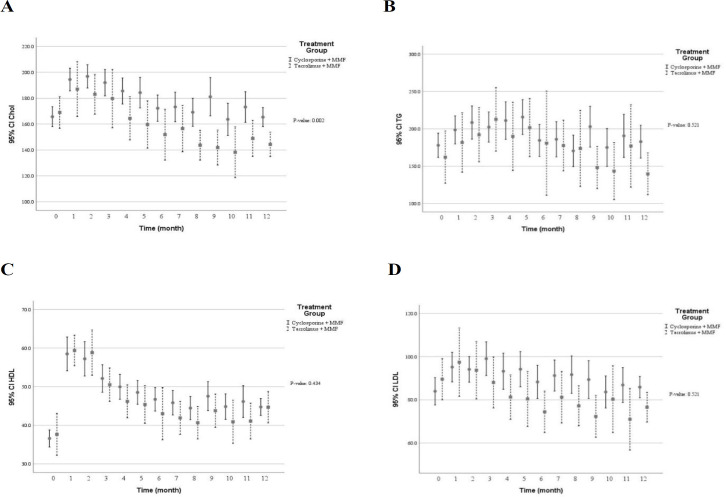
The lipid profile within the first year after kidney transplantation in the patients received tacrolimus *vs* cyclosporine (A) total blood cholesterol concentration (mg/dL) (B) blood triglyceride concentration (mg/dL) (C) blood high-density lipoprotein concentration (mg/dL) (D) blood low-density lipoprotein (mg/dL). Significantly lower total cholesterol levels were associated with tacrolimus treatment by means of the multivariable predictive analysis. (*p* ˂ 0.05); MMF: mycophenolate mofetil

**Figure 6:**
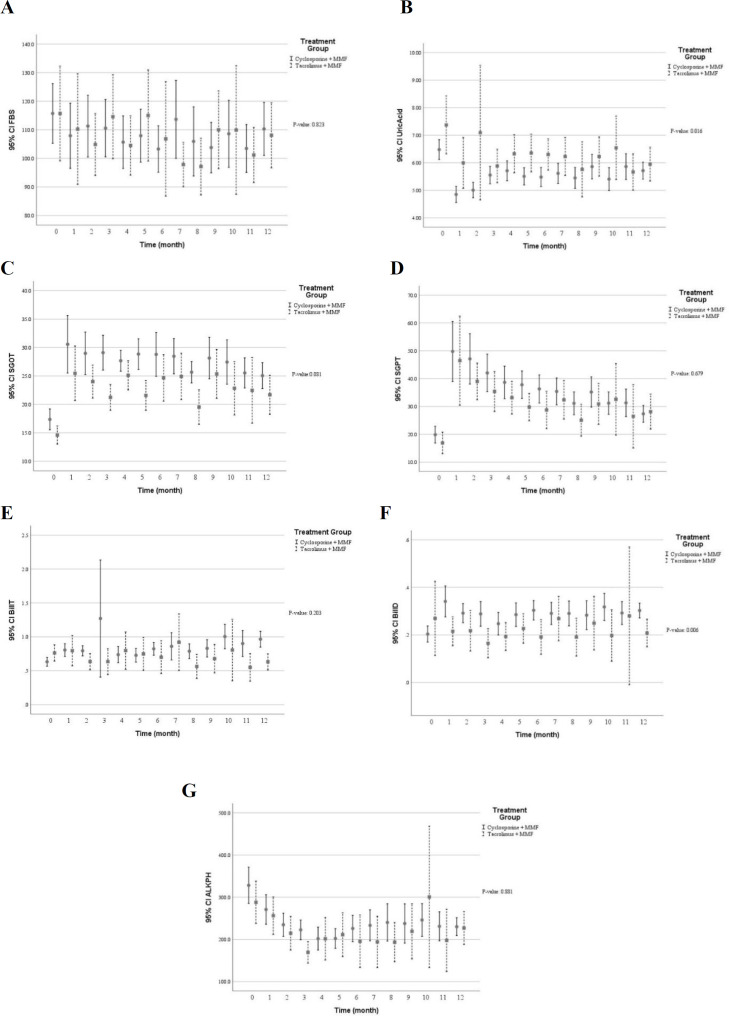
The glucose and liver function profile within the first year after kidney transplantation in the patients received tacrolimus *vs* cyclosporine. (A) fasting blood sugar (mg/dL) (B) serum uric acid concentration (mg/dL) (C) serum glutamic-oxaloacetic transaminase concentration (u/L) (D) serum glutamic-pyruvic transaminase concentration (u/L) (E) total serum bilirubin concentration (mg/dL) (F) direct serum bilirubin concentration (mg/dL) (G) blood alkaline phosphatase concentration (mg/dL). Significant lower levels of direct bilirubin and significant higher levels of serum uric acid were associated with tacrolimus treatment by means of the multivariable predictive analysis. (*p* ˂ 0.05); MMF: mycophenolate mofetil

**Figure 7:**
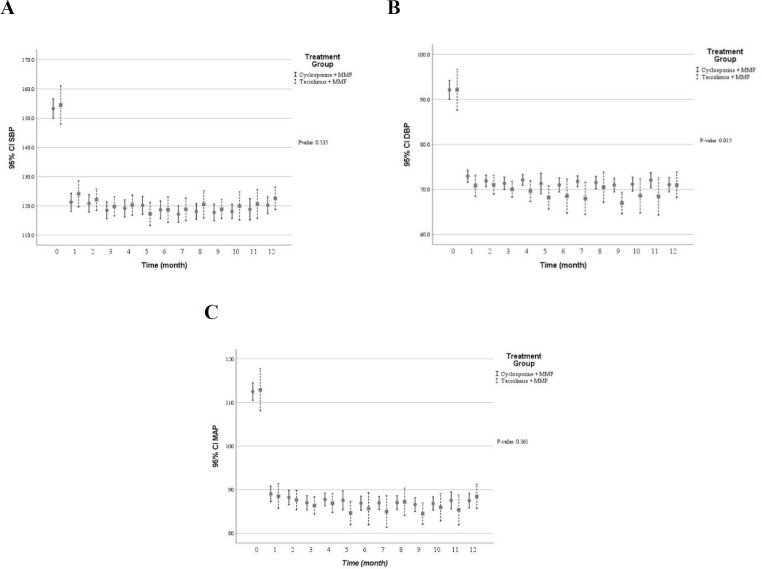
The blood pressure profile within the first year after kidney transplantation in the patients received tacrolimus *vs* cyclosporine (A) systolic blood pressure (mmHg) (B) diastolic blood pressure (mmHg) (C) mean arterial pressure (mmHg). The multivariable predictive analysis demonstrated significant lower diastolic blood pressure in patient receiving tacrolimus. (*p* ˂ 0.05); MMF: mycophenolate mofetil

Table 3 shows multivariate predictive analysis of adjusted clinical and laboratory changes following the CNI switching. Accordingly, the patients with CNI switching to TAC showed lower creatinine clearance (*p* = 0.050), reduced eGFR (*p* = 0.073), and increased serum creatinine (*p* = 0.195). Those results indicated that diminished graft function in patients switched TAC. However, no statistically significant difference was found. Patients whose medication was converted from CsA to TAC showed significant decrease in diastolic blood pressure (*p* = 0.015), levels of serum total cholesterol (*p* = 0.002) and direct bilirubin (*p* = 0.006) as well as number of white blood cells (*p* = 0.004). Conversely, higher levels of serum potassium (*p* = 0.001) and uric acid (*p* = 0.016) were found following the CNI changed. Using multivariate predictive analyses models, the time course of several clinical and laboratory parameters within the first year of kidney transplantation in the patients converted to TAC versus the patients on CsA are depicted in Figures 2 to 7.

**Table 1:** Comparison of the baseline characteristics between the study groups

**Characteristic**	**CsA + MMF** **(n = 125)**	**TAC + MMF** **( n = 41)**	**P-value**
**Demographics**			
Age, year	41.8 ± 14.2	38.9 ± 11.3	0.199
Male sex, n (%)	93 (74.4)	29 (70.7)	0.644
**Cause of ESRD**			0.107
Unknown, n (%)	32 (25.6)	3 (7.3)	
Hypertension, n (%)	47 (37.6)	17 (41.5)	
Diabetes mellitus, n (%)	8 (6.4)	1 (2.4)	
Hypertension and Diabetes mellitus, n (%)	20 (16.0)	7 (17.1)	
Urinary reflux, n (%)	2 (1.6)	1 (2.4)	
Hypertension and reflux, n (%)	2 (1.6)	1 (2.4)	
Polycystic kidney disease, n (%)	3 (2.4)	2 (4.9)	
Nephrolithiasis, n (%)	3 (2.4)	3 (7.3)	
Miscellaneous, n (%)	8 (6.4)	6 (14.6)	
Diabetes mellitus, n (%)	28 (22.4)	8 (19.5)	0.697
Hypertension, n (%)	69 (55.2)	27 (65.9)	0.231
**Pre-transplantation measures ᵅ**			
Weight ( kg)	66.4 ± 13.9	70.3 ± 17.1	0.143
Systolic blood pressure (mmHg)	153.2 ± 18.8	154.4 ± 20.9	0.731
Diastolic blood pressure (mmHg)	92.1 ± 11.6	92.1 ± 14.3	0.999
MAP (mmHg)	112.5 ± 11.3	112.9 ± 15.3	0.858
WBC (×10ˆ3/μL)	8.22 ± 4.26	8.22 ± 4.96	0.995
Hemoglobin (gr/dL)	11.4 ± 2.3	11.5 ± 2.1	0.781
Hematocrit (%)	35.8 ± 6.7	35.9 ± 5.4	0.972
Platelets (×10ˆ3/μL)	209.2 ± 85.1	205.8 ± 73.1	0.818
Sodium (mEq/L)	138.4 ± 4.5	136.4 ± 3.4	0.956
Potassium (mEq/L)	5.17 ± 0.84	5.00 ± 0.93	0.226
Calcium (mg/dl)	8.70 ± 1.35	8.88 ± 0.96	0.442
Phosphorus (mg/dL)	5.28 ± 1.58	4.98 ± 1.40	0.281
AST (U/L)	17.36 ± 10.35	14.60 ± 4.68	0.115
ALT (U/L)	19.87 ± 14.92	16.91 ± 9.00	0.364
Total bilirubin (mg/dL)	0.63 ± 0.31	0.76 ± 0.27	0.07
Direct bilirubin (mg/dL)	0.20 ± 0.16	0.26 ± 0.36	0.194
Alkaline phosphatase (mg/dL)	328.2 ± 243.0	287.9±159.1	0.322
Fasting blood sugar (mg/dL)	115.7 ± 59.1	115.7 ± 52.5	0.999
Cholesterol (mg/dL)	165.6 ± 43.8	168.9 ± 39.8	0.666
Triglyceride (mg/dL)	177.9 ± 91.4	162.1 ± 110.7	0.362
HDL (mg/dL)	36.6 ± 10.6	37.6 ± 12.5	0.679
LDL (mg/dL)	84.0 ± 30.3	89.5 ± 22.0	0.409
Uric acid (mg/dL)	6.47 ± 2.02	7.37 ± 3.33	0.041
Serum Creatinine (mg/dL)	8.47 ± 3.34	7.47 ± 2.87	0.09
eGFR (ml/min)	8.22 ± 5.64	9.40 ± 5.60	0.246
**Early Post-transplantation measures ᵇ**			
IV Methylprednisolone dose (mean ± SD;mg/kg)	24.4 ± 12.4	22.9 ± 11.1	0.49
MAP (mmHg)	89.0 ± 9.3	88.5 ± 6.9	0.799
Serum Creatinine (mg/dL)	6.04 ± 2.71	6.47 ± 3.07	0.391
Serum Creatinine Clearance (ml/min)	66.15 ± 26.22	67.82 ± 20.93	0.757
eGFR (ml/min)	60.05 ± 22.79	54.45 ± 18.51	0.228
Prednisolone dose (mean ±SD;mg/day)	50.3 ± 17.7	45.3 ± 15.0	0.111
Cyclosporine dose (mean ± SD;mg/day)	347.3 ± 99.3	310.3 ± 159.7	0.169
Mycophenolate mofetil dose (mean ± SD;mg/day)	1637±379.02	1465.0 ± 354.8	0.011

CsA: Cyclosporine; MMF: Mycophenolate mofetil; TAC: Tacrolimus; ESRD: End-stage renal disease; MAP: Mean arterial pressure; WBC: White blood cell; AST: Aspartate aminotransferase; ALT: Alanine aminotransferase; HDL: High-density lipoprotein; LDL: Low-density lipoprotein; eGFR: Estimated glomerular filtration rate.

a: All the data were related to the before transplantation.

b: All the data were related to time of discharge after transplantation.

**Table 2:** The clinical & laboratory outcomes following kidney transplantation

**Parameter**	**CsA +MMF** **(n = 125)**	**TAC +MMF** **(n = 41)**	***P*** **-value**
**Short-term ᵅ**			
New-onset hypertension, n (%)	5 (71.4)	4 (100)	0.491
NODAT, n (%)	9 (9.3)	7 (21.2)	0.12
Hypertension treatment goal reached ᵇ, n (%)	108 (86.4)	37 (90.2)	0.601
Serum Creatinine (mg/dL)	1.44 ± 0.53	1.57 ± 0.41	0.165
Serum Creatinine Clearance (ml/min)	67.37 ± 17.02	66.47 ± 20.37	0.783
eGFR (ml/min)	61.70 ± 14.73	56.66±17.82	0.074
MAP (mmHg)	87.77 ± 5.25	86.96	0.413
Mean Prednisolone dose (mean ± SD;mg/day)	17.7 ± 4.1	15.8 ± 4.4	0.013
Mean CNI dose (mean ± SD;mg/day)	224.9 ± 35.8	4.61 ± 1.55	-
Mean Mycophenolate mofetil dose (mean ± SD;mg/day)	1494.71 ± 304.73	1371 ± 193.8	0.003
BPAR, n (%)	1 (0.8)	0 (0)	0.566
Graft loss, n (%)	1 (0.8)	0 (0)	0.566
All-causes mortality, n (%)	0 (0)	3 (7.3)	0.002
**Long-term ͨ**			
BPAR , n (%)	1 (0.8)	0 (0)	0.566
Graft loss, n (%)	1 (0.8)	0 (0)	0.566
All-causes mortality, n (%)	1(0.8)	6(14.6)	<0.001
**Type of antihypertentsive medication**			
Hypertension treatment			0.042
ACEI/ARB, n (%)	37 (29.6)	21 (51.2)	
Non-ACEI/ARB, n (%)	65 (52.0)	15 (36.6)	
No medication, n (%)	23 (18.4)	5 (12.2)	

CsA: Cyclosporine; MMF: Mycophenolate mofetil; TAC: Tacrolimus; NODAT: New onset diabetes mellitus after transplantation; eGFR: Estimated glomerular filtration rate; MAP: Mean arterial pressure; BPAR: Biopsy-proven acute rejection; ACEI: Angiotensin-converting-enzyme inhibitors ARB: Angiotensin II receptor blocker.

a: The short term outcome corresponds to the values within the first year post transplantation.

b: BP <130/80 (mmHg).

c: The long term outcome corresponds to the values within the 2-8years; (median follow up of 5 years) post transplantation.

**Table 3:** Multivariable predictive model for clinical and laboratory changes based on the type of CNI treatment ͣ.

**Characteristic**	**Beta**	**Standard error**	**95% confidence interval**	**P-value**
Systolic blood pressure (mmHg)	0.954	1.538	-2.061, 3.970	0.535
Diastolic blood pressure (mmHg)	-2.015	0.83	-3.643, -0.388	0.015
MAP (mmHg)	-0.882	0.966	-2.776, 1.012	0.361
WBC (×10ˆ3/μL)	-0.94	0.324	-1.575, -0.304	0.004
Hemoglobin (gr/dL)	-0.166	0.286	-0.728, 0.396	0.562
Hematocrit (%)	-1.193	1.084	-3.319, 0.931	0.271
Platelets ( number;×10ˆ3/μL)	3.693	8.202	-12.382, 19.770	0.652
Sodium (mEq/L)	-0.227	0.326	-0.867, 0.411	0.485
Potassium (mEq/L)	0.191	0.055	-0.299, 0.083	0.001
Calcium (mg/dL)	0.003	0.066	-0.126, 0.133	0.953
Phosphorus (mg/dL)	0.135	0.097	-0.325, 0.054	0.163
AST (U/L)	-2.796	1.601	-5.935, 0.343	0.081
ALT (U/L)	-1.831	4.431	-10.516, 6.853	0.679
Total bilirubin (mg/dL)	-0.215	0.169	-0.547, 0.116	0.203
Direct bilirubin (mg/dL)	-0.104	0.037	-0.178, -0.029	0.006
Alkaline phosphatase (mg/dL)	-2.743	18.392	-38.792, 33.304	0.881
Fasting blood sugar (mg/dL)	1.194	5.348	-9.287, 11.676	0.823
Cholesterol (mg/dL)	-16.679	5.389	-27.241, -6.116	0.002
Triglyceride (mg/dL)	-8.164	12.729	-33.114, 16.785	0.521
HDL (mg/dL)	-1.997	2.552	-7.000, 3.006	0.434
LDL (mg/dL)	-3.777	5.887	-15.315, 7.761	0.521
Uric acid (mg/dL)	0.574	0.237	0.108, 1.040	0.016
Serum creatinine (mg/dL)	0.111	0.086	-0.057, 0.280	0.195
eGFR (ml/min)	-4.154	2.138	-8.369, 0.388	0.073
Urea (mg/dL)	2.381	3.168	-3.827, 8.590	0.452
Creatinine clearance (ml/min)	-4.756	2.429	-9.158, 0.005	0.050

MAP: Mean arterial pressure; WBC: White blood cells number; AST: Aspartate aminotransferase; ALT: Alanine aminotransferase; HDL: High-density lipoprotein; LDL: Low-density lipoprotein; eGFR: Estimated Glomerular Filtration Rate.

a This is adjusted for age, sex, baseline weight, diabetes mellitus, hypertension, antihypertensive regimen, blood group and the baseline value for every single variable. Cyclosporine continuation was considered as the reference so the beta shows the effect of the conversion to Tacrolimus.

## Discussion

According to current literature the conversion from CsA to TAC following kidney transplantation can be utilized in one of the following situations: first, due to appearance of renal or extra-renal adverse effects of CsA including acute or chronic nephrotoxicity, hypertension, dyslipidemia and etc; second, to minimize the severity of CAN; third, in the case of acute rejection and forth; pre-emptive late conversion in stable kidney grafts to maintain the graft function (1, 4-6, 13-23). However, to the best of our knowledge, this is the first study conducted on early CNI switching from CsA to TAC in kidney graft with acceptable function.

The timing of CNI switching to TAC was very variable among different studies. However, majority of studies demonstrated beneficial effects of the switching on graft function regardless of onset time of conversion.

As an example for studies with early conversion, Shihab *et al*. showed improved creatinine clearance, serum creatinine, and blood urea nitrogen in patients with CAN converted to tacrolimus at ≥ 3 months after transplantation (14). Chamienia *et al*. evaluated the conversion to TAC at ≥ 6 months after transplantation in the patients with cyclosporine-induced nephrotoxicity and other cyclosporine-related adverse effect. They reported improved graft function (16). In the context of refractory rejection, Maroun *et al.* demonstrated a high rate of graft salvage and improved kidney function following early conversion to TAC at a median of 92 days after-transplantation (21). Morales *et al.* reported the successful control of steroid-resistant acute rejection and reduced graft loss and recurrent rejection in patients converted early to TAC (22). In addition, Jordan *et al.* showed that patients with ongoing biopsy-proven acute rejection (BPAR) experienced improved graft function following early conversion to TAC at a median of 2 months after transplantation (23).

As a study with late-onset conversion to TAC, Marcard *et al.* found a significant improvement of graft function in patients with CAN undergone such a conversion at 18-21 months after transplantation (13). Artz *et al.* studied the efficacy of conversion to TAC *vs* CsA continuation in stable grafts at ≥ 1 year after transplantation. The results showed improved graft function following such late conversion (19). According to Plischke *et al.* and Krejci *et al*. studies, patients with stable graft receiving switched TAC showed ameliorated graft function when the onsets of switching were after ≥ 5.7 and 8 years of transplantation, respectively (18, 20).

In contrast to the aforementioned studies, we found that early pre-emptive conversion to TAC in normally functioning graft at time of hospital discharge was not associated with superior graft function in comparison to patients on CsA. Indeed, multivariate predictive analyses showed lower creatinine clearance and diminished eGFR as well as higher levels of serum creatinine in patients who switched to TAC. However, no statistically significant difference was found. 

In concordance with our results, Jevnikar *et al.* (2008) found no superiority in terms of graft function following conversion to TAC in patients with CAN (4). Similarly, Margareiter *et al* (2005) and Usukui *et al.* (2018) showed no statistically significant difference of graft functions between patients switched to TAC and patients continued with CsA treatment (1,5).

Despite the relative consensus about benefits of conversion to TAC on graft function, the efficacy of this switching on graft survival is controversial. In Chamienia *et al.* (2006) study, institution of switched TAC resulted in higher graft survival within a 6-month follow-up (16). Concordant findings were reported by Usuki *et al.* following 2 years after conversion to TAC in patients with prior cyclosporine-induced adverse effects (1). Conversely no improved 2-year graft survival was reported in patients with stable kidney graft randomly assigned to switched TAC (19). As shown by Artz* et al. *(2004) (19), we showed that no significant difference between the patients receiving TAC and those treated with CsA in terms of one-year graft loss and BPAR. Some authors evaluated long-term graft function and graft survival following conversion to tacrolimus. Shihab *et al.* (2008) demonstrated superior graft function following 5 years subsequent to switching from CsA to TAC in CAN. However, improved 5-year rates of graft loss and rejection were not reported (14). Through a follow-up of 30 months, Markard *et al* (2008) reported significantly better graft function in patients with CAN converted from CsA to TAC (13). In contrast, Jevnikar *et al. *(2008) showed no superiority in terms of graft function, graft loss, and BPAR within 5 years following the conversion (4). According to our results, BPAR and graft loss were not significantly different following CsA continuation *vs* early conversion to TAC in normally functioning graft with a median 5 years of follow-up.

The current literature showed no increase in short-term and long-term patient survival following CNI switching from CsA to TAC in different clinical settings. For example, Shihab *et al.* (2008) and Jevnikar *et al. *(2008) showed no superior patient survival after 5 years of the conversion (4, 14). Artz *et al.* (2004) reported similar finding following 2 years of CNI switching in patients with stable kidney grafts randomly assigned to TAC and CsA treatments (19). In contrast, we reported higher rates of all-causes mortality in the patients switched to TAC following both short-term (1 year) and long term (median 5 years) follow-up.

The effect of CNI switching to TAC on cardiovascular risk profile is widely controversial. Artz *et al.* demonstrated lower Framingham risk scores in patients converted to TAC (19). Significant decrease in serum levels of total cholesterol, LDL cholesterol, and triglycerides has been reported in the patients received TAC instead of CsA (5, 6). However, there are some studies with discordant results indicating no significant improved lipid profile following conversion to TAC (1, 19). Interestingly, Shihab *et al.* reported that continuation of CsA was associated with lower incidence of new-onset hypercholesterolemia (14). TAC therapy associated with poor glycemic control in diabetic recipients and higher incidence of NODAT (24, 25, 26). In contrast to such belief, Chamienia *et al.* report no *de novo* case of diabetes following conversion to TAC (16). In addition, Krejci* et al.* demonstrated that there was no significant difference in fasting plasma glucose between the patients converted to TAC and those maintained on CsA (18). According to previous studies, lower levels of blood pressure are expected following tacrolimus therapy (5). However, Markard *et al.* documented no significant changes in mean arterial blood pressure following conversion to TAC (13). Krejci *et al.* reported significant decrease in diastolic blood pressure but not-significant reduced systolic blood pressure in patients undergone switching to TAC (18). In addition, Shihab *et al.* found higher incidence of new-onset of hypertension in patients maintained on CsA; but the finding was not statistically significant (14). There is no documentation showing superiority of switching to tacrolimus to achieve goals of blood pressure control. However, Krejci *et al.* reported an important decrease in number of antihypertensive medications subsequent to conversion to tacrolimus (18).

According to our results the patients treated with TAC instead of CsA showed reduced serum levels of total cholesterol, LDL cholesterol, and triglycerides. However, multivariable predictive analyses confirmed that only decrease in total cholesterol was statistically significant. Conversion to TAC was associated with increased incidence of NODAT which was statistically not significant. Similarly, the patients who switched to TAC showed non-significant higher levels of fasting blood sugar. Following conversion to TAC significant decrease in diastolic blood pressure but non-significant in systolic blood pressure were found. In addition, MAP was associated with a non-significant decrease in the patients treated with TAC. Higher incidence of new-onset hypertension as well as more successful control of blood pressure were seen in the patients who underwent TAC treatment; but these findings were not statistically significant. Similar to finding reported by Marcard *et al.* (13), we found that most frequently-used antihypertensive medications were ACEI/ARB in the patients receiving TAC. Non-ACEI/ARB medications were used most commonly in the patients who were maintained on CsA treatment. Collectively cardiovascular risk factors in our patients who received TAC were better than those patients continued with CsA therapy. The higher rates of short- and long-term all-causes mortality in the context of improved cardiovascular risk profile were remained unexplained.

We found significant decrease in serum level of uric acid following CNI switching to TAC. No similar findings were reported elsewhere and underling mechanisms have not been explained. Similar to Krejci *et al.* our results showed a non-significant decreased of hepatic aminotransferases in the patients treated with TAC (18). In addition, decreased serum levels of alkaline phosphatase, total bilirubin, and direct bilirubin were found. However, statistically significance was limited to reduced serum level of direct bilirubin. Studies evaluating lower incidence of non-alcoholic fatty liver disease (NAFLD) in the patients receiving TAC can be an era for future research.

*Study limitations*

Because of retrospective design of present study, random assignment of the patients to the study groups was out of authors’ authority. In addition, no data on patients’ HLA typing and advanced screenings such as flow cytometric panel reactive antibody was available. The result of the virtual cross-match between donors and recipients of kidney allograft had not been specified. The plasma CNIs trough levels were not available.

## Conclusion

Early pre-emptive conversion from CsA to TAC in normally functioning grafts was not associated with improved graft and patient outcomes. The continuation of initial CsA might be a good option when the graft function is acceptable and the adverse effects are absent.
